# How the immune system spots tumors

**DOI:** 10.7554/eLife.04476

**Published:** 2014-09-24

**Authors:** Yasutaka Okabe, Ruslan Medzhitov

**Affiliations:** 1 **Yasutaka Okabe** is in the Department of Immunobiology, Yale University School of Medicine, New Haven, United States; 2 **Ruslan Medzhitov** is an *eLife* reviewing editor, and is in the Department of Immunobiology, Yale University School of Medicine, New Haven, United Statesruslan.medzhitov@yale.edu

**Keywords:** innate immunity, Dectin-1, NK cell, IRF, cancer, mouse

## Abstract

The receptor protein Dectin-1 recognizes structures found on cancerous cells, and then triggers an anti-tumor immune response.

**Related research article** Chiba S, Ikushima H, Ueki H, Yanai H, Kimura Y, Hangai S, Nishio J, Negishi H, Tamura T, Saijo S, Iwakura Y, Taniguchi T. 2014. Recognition of tumor cells by Dectin-1 orchestrates innate immune cells for anti-tumor responses. *eLife* **3**:e04177. doi: 10.7554/eLife.04177

**Image** Cancerous tumors form and spread much more easily in the lungs of mice that cannot produce Dectin-1 (bottom)

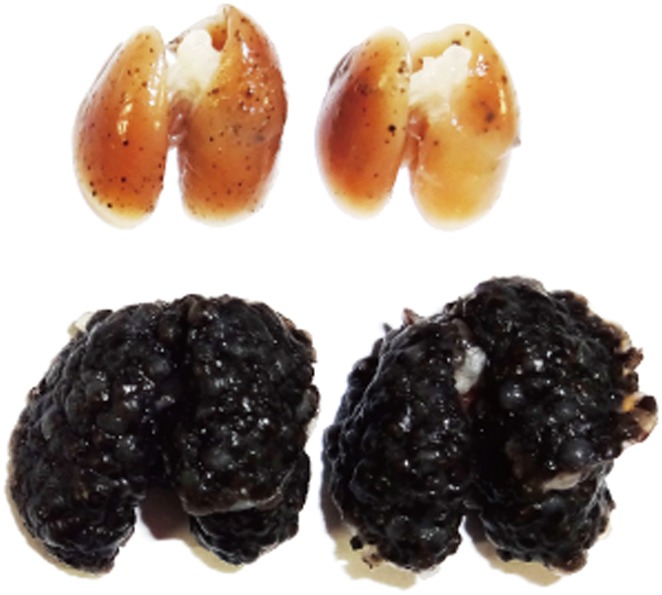

The immune system is best known for its role in defending the body against microbial infections. People carrying mutations that affect the immune system are, therefore, highly susceptible to infectious diseases and usually require continuous care to avoid infections. However, it has also been proposed that the immune system can eliminate cancer cells. This idea, known as immune surveillance, is several decades old, but there is little experimental evidence to support it. In large part, this is because the mechanisms used by the body to recognize cancer cells remain poorly understood.

A key feature of the immune system is its ability to distinguish between pathogens (‘non-self’) and the body's own cells (‘self’). Immune cells detect certain components that are found in many microorganisms, such as the complex carbohydrates that make up bacterial and fungal cell walls. Because these components are unique to microbes, detecting them allows the innate immune response—the body's first line of defence—to identify the cell they came from as ‘non-self’ and potentially harmful, and then act to neutralize the threat.

The carbohydrates are recognized by the so-called pattern-recognition receptors, such as Toll-like receptors and C-type lectin receptors, which are found in two types of immune cell—dendritic cells and macrophages (Takeuchi and Akira, 2010). Pathogens cannot easily evade detection because the components that are recognized by the receptors are essential for microbial fitness and survival.

This approach cannot be used to detect tumors because they develop from the host's own cells. Rather, in order to perform immune surveillance the immune system has to be able to distinguish tumor cells from normal cells. One way that this would be possible is if cancer cells produce signals that can be detected by the immune system. Clearly, if such signals exist, they would be the first feature to be lost by the cancer cells as they evolve under the selection pressure imposed by immune surveillance. Unless, that is, getting rid of these signals comes with a significant cost to the cancer cell.

Now, in *eLife*, Tadatsugu Taniguchi of the University of Tokyo and co-workers—including Shiho Chiba, Hiroaki Ikushima and Hiroshi Ueki as joint first authors—report evidence that immune surveillance does indeed rely on this strategy. They report that the pattern-recognition receptor Dectin-1 plays a crucial role in recognizing a process called tumor cell-associated glycosylation and in initiating an anti-tumor innate immune response (Chiba et al., 2014).

Glycosylation is a reaction where a carbohydrate molecule called a glycan is added to a protein. Altered glycosylation is a universal feature of tumor cells (Varki et al., 2009). By increasing where and how much glycosylation occurs on their surface, many tumor cells develop an advantage over other cells when migrating to and spreading through other organs.

Chiba et al. found that two proteins play crucial roles in suppressing the spread of cancer: Dectin-1 and IRF5 (Figure 1). Dectin-1 recognizes molecules called β-glucans that are found on fungal cell walls (Brown, 2006). When Dectin-1 recognizes its target, the transcription factor IRF5 activates innate immunity in response (del Fresno et al., 2013). Chiba et al. now show that Dectin-1 also recognizes particular glycosylation associated structures called N-glycan structures, which are highly expressed in several tumor cell lines—but not in non-cancerous cells. When N-glycan structures are detected, Dectin-1 triggers an anti-tumor immune response.

**Figure 1.**
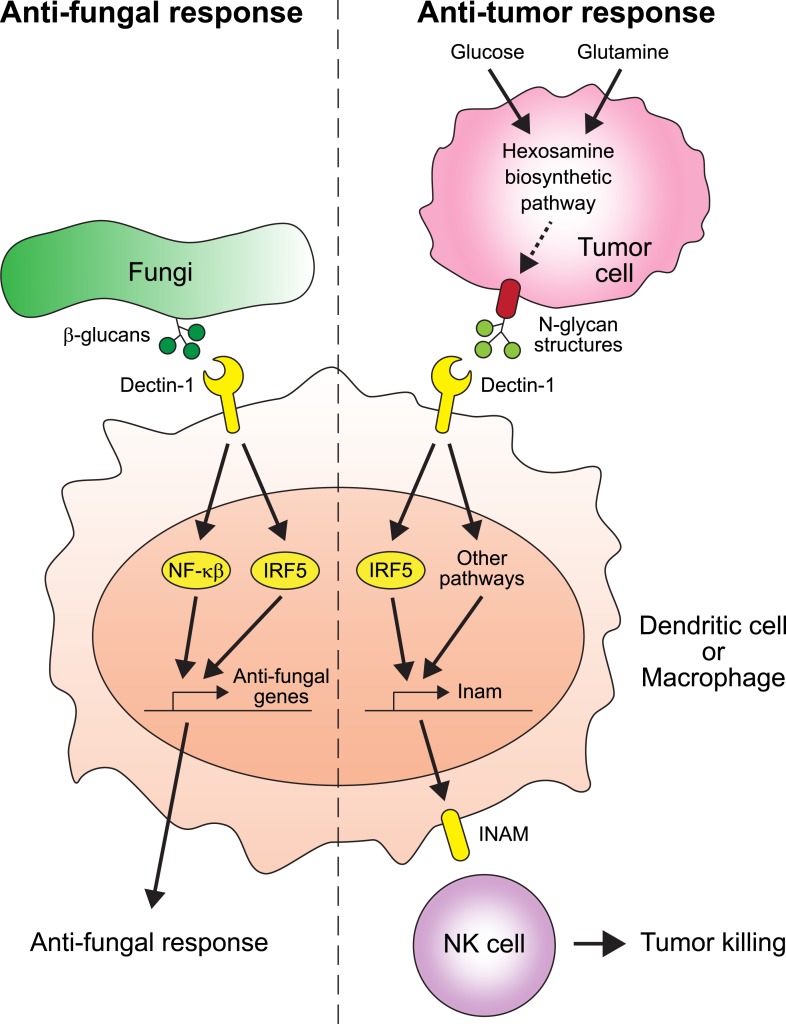
The receptor protein Dectin-1 plays an important role in the anti-fungal and anti-tumor responses of the innate immune system. Left: Dectin-1 is expressed on dendritic cells and macrophages, and recognizes molecules called β-glucans (dark green circles) on fungal cell walls. This recognition triggers the signaling pathways that activate transcription factors including IRF5 and NF-κβ (del Fresno et al., 2013), which cause anti-fungal genes to be expressed. Right: Chiba et al. found that Dectin-1 can also recognize N-glycan structures expressed on certain tumor cell lines. Recognizing these tumor cell signals activates IRF5 and perhaps other unidentified pathways, which causes tumoricidal action by natural killer (NK) cells. At least part of the mechanism for natural killer cell mediated tumor killing involves increasing the expression of the Inam gene. It would be interesting in the future to determine if Dectin-1 recognizes N-glycan structures as a proxy for the increased activity of the hexosamine biosynthetic pathway in tumor cells.

Natural killer (NK) cells are lymphocytes (or white blood cells) that can kill cancer cells and so could play an important role in immune surveillance (Vivier et al., 2012). Chiba et al. found in their experimental model that natural killer cells are indeed required for the elimination of tumor cells. Furthermore, natural killer cells rely on Dectin-1 expressed on dendritic cells and macrophages in order to kill tumor cells; the findings therefore reveal a new functional link between dendritic cells, macrophages and natural killer cells.

Chiba et al. have taken an important step towards understanding how the anti-tumor immune response works. As is normal for a brand-new finding, this work raises a number of important questions. The level and/or the specific structural patterns of glycosylation on tumor cells appears to be important for the fitness of cancer cells. Therefore, an immune surveillance system based on cell surface glycan recognition may have evolved to prevent cancer cells themselves evolving in a way that enables them to evade the anti-tumor immune response. In this regard, it would be important to know whether Dectin-1 is activated by either an altered structure or increased level of N-glycans on tumor cells.

It is possible that the total level or specific structural variants of cell surface N-glycans may reflect the metabolic state of the cell. For example, the hexosamine biosynthetic pathway—which is used by cells to produce a particular type of sugar—is more active than normal in some cancers and can affect the glycosylation patterns of cell surface proteins (Wellen et al., 2010). It would be very interesting to investigate whether Dectin-1 (and other C-type lectins) evolved to detect the increased activity of the hexosamine biosynthetic pathway through its effect on cell surface N-glycans, as a proxy for cancer cells (Figure 1).
